# A facile synthesis of silver nanoparticles using Woodfordia fruticosa flower extracts for certain bacteria inhibition applications

**DOI:** 10.1016/j.heliyon.2025.e42125

**Published:** 2025-01-21

**Authors:** Venkatachalam Dhanapal, Pushparaju Subhapriya, Karpaganathan Arangarajan, Arumugam Jeevanantham, Perumal Sudhakar, Velappan Kandavelu, K.S. Nivedhitha, M.A. Umarfarooq, N.R. Banapurmath, Irfan Anjum Badruddin, Muhammad Nasir Bashir, Muhammad Mahmood Ali

**Affiliations:** a PG and Research Department of Chemistry, Sri Ramakrishna Mission Vidyalaya College of Arts and Science, Coimbatore, 641 020, Tamil Nadu, India; b Department of Chemistry, Bannari Amman Institute of Technology, Sathyamangalam- 638 401 Erode Dt, Tamil Nadu, India; c Centre for Material Science, KLE Technological University, Hubballi, Karnataka-580031, India; d Centre for Research Impact and Outcome, Chitkara University, Punjab, 140401, India; e Mechanical Engineering Department, College of Engineering, King Khalid University, Abha 61421, Saudi Arabia; f Multi-Scale Fluid Dynamics Lab, Department of Mechanical Engineering, Yonsei University, Seoul, 120-749, Republic of Korea; g Department of Mechatronic Engineering, Atlantic Technological University Sligo, Ash Lane, F91 YW50, Sligo, Ireland

**Keywords:** Woodfordia fruticosa, Silver nanoparticles, Anti-bacteria activity, Energy dispersive X-ray spectroscopy

## Abstract

The present research focused on extraction of bioactive compounds from Woodfordia fruticosa flower (WF) using ethanol, methanol and ethyl acetate as solvents and the development of silver nanoparticles using these extracts for inhibition of *Staphylococcus aureus* (*S. aureus*), *Klebsiella pneumoniae* (*K. pneumoniae*), *Escherichia coli* (*E. coli*), and Bacillus cereus (B. cereus) bacteria. The functional groups of bioactive compounds present in the solvent extracts were characterized using Fourier transform infrared spectroscopy (FT-IR). The morphological features and formation of silver nanoparticles (10–30 nm, by the reduction of solvent extracts) were evaluated using scanning electron microscopy (SEM) and ultraviolet–visible (UV–Vis) spectroscopy, respectively. The elemental compositions of the synthesized nanoparticles were analyzed using energy-dispersive X-ray spectroscopy (EDX). The inhibition efficiency of the alcoholic and ester extracts of WF and the synthesized silver nanoparticles were evaluated and compared to Moxifloxacin. The results revealed that the synthesized silver nanoparticles demonstrated enhanced bacterial inhibition efficiency compared to the unprocessed ethanol, methanol, and ethyl acetate extracts of WF, and Moxifloxacin.

## 1 Introduction

Bioactive compounds obtained from plants play an important role both in traditional and modern medication. Many plants contain therapeutic micro and macro active compounds that facilitate the disintegration of microbes and pathogens [1]. The efficacy of antibiotics used today has been increasingly challenged by multidrug-resistant pathogens. These disease-causing microorganisms have developed numerous protective mechanisms and exhibit greater tolerance against applied drugs. Hence, this noticeable failure of therapeutic agents against numerous microbes forced greater [2,3]. Bioactive compounds derived from various plants have demonstrated impressive inhibitory efficiency against diverse bacteria [[4], [5], [6], [7]]. One such plant is WF, whose derived compounds have been shown to be effective for diverse bacteria inhibition. Comprehensive research has been reported in the literature on the extracts of WF plant and flower parts. For instance, Dubey et al. [8] reported that the leaf extract of WF has the ability to control multidrug-resistant bacteria, and the crude leaf and flower extracts showed no host toxicity in human lymphocytes. In addition, Mukaratirwa-Muchanyereyi et al. [9] synthesized silver nanoparticles using plant extracts from the aerial parts of Erythrina abyssinica. Naveed et al. [10] synthesized silver nanoparticles through a green synthetic route and applied them for antibacterial purposes. Urnukhsaikhan et al. studied the antibacterial activity of silver nanoparticles synthesized using Carduus crispus plant extract [11]. The antioxidant, free radical scavenging, and bacterial inhibition activity of bioactive compounds against gram-positive and gram-negative bacteria were due to the presence of phenolic residues in the flower extract [12]. Due to these widespread applications, the flowers and other parts of WF are in high demand, both in national and international markets to prepare herbal medicine for the ailments of many diseases [13]. Additionally, extracts (aqueous and organic solvents) of WF plant parts have been extensively used for the biogenic synthesis of metal nanoparticles. Although well-defined nanoparticles can be synthesized through various physicochemical methods, the phyto-synthetic method was predominantly used by many researchers [14] due to its cost-effectiveness, less energy consumption, and harmless discharge of residual waste into the environment [15,16].

In addition to antibacterial activity, bioactive compounds derived from plants have been found useful for wound healing [17] anti-inflammatory and antimicrobial [18] and antifertility activities [19]. Furthermore, bioactive compounds obtained from WF have shown hepatoprotective, cardioprotective, antioxidant, anti-tumor, antihyperlipidemic, anthelmintic, antihyperglycemic, immunomodulatory, antiulcer, and analgesic activities [20]. The noteworthy clinical activities of these bio-extracts are due to numerous phytochemicals namely flavonoids, terpenoids, tannins, and phenolic compounds [21]. In addition to these, Grover and Patni [22] reported that a dark yellowish-brown dye obtained from WF flowers was used for dyeing cotton-jute mix and pure cotton fabrics. Yu et al. [23] isolated three phenolic compounds namely (±)-woodfordiamycin, woodfordic acid, and rhamnetin 3-O-(6-galloyl)-β-d-glucopyranoside for the skin disease caused by certain fungi. Kushlani et al. [24] reported antifertility activity from dried WF flower extracts in female albino rats. Additionally, a tannin dimer isolated from dried WF flowers exhibited antitumor activity in human cells [25]. Rose and Prasad [26] found that bioactive compounds isolated from the stem bark of WF exhibited analgesic activity in albino rats. Khera and Bhatia [27] reported that methanolic flower extracts of WF lowered lipid parameters in mice fed a high-cholesterol diet. Sengupta et al. [28] investigated the anthelmintic activity of methanol and petroleum ether extracts derived from dried WF flowers against the Indian earthworm Pheretima posthuma. However, the present study focused on extracting bioactive compounds using solvents such as ethanol, methanol, and ethyl acetate, and the biogenic synthesis of silver nanoparticles from silver nitrate using these extracts, and their physicochemical characterization. Subsequently, the alcoholic extracts were characterized for their antibacterial activity against *S. aureus*, *K. pneumoniae*, *E. coli*, and *B. cereus*.

## 2 Materials and methods

### 2.1 Chemicals

The solvents ethanol, methanol, and ethyl acetate (Merck) were received and purified before use, while the silver nitrate (Merck) was used without further purification. Nutrient agar (Oxoid) was used as received. The bacterial strains *S.aureus, K. pneumonia, E.coli,* and *B.cereus* were obtained from the Centre for Bioscience and Nano-science Research, Coimbatore, India. Mueller-Hinton agar (Himedia Laboratories, Mumbai, India) and Moxifloxacin (Chromo Labs, India) were used as received. Glassware were washed many times using deionized hot water and dried in a hot air oven for 5 h before use.

### 2.2 Characterization techniques

The FT-IR spectra of bioactive and nanoparticles were recorded on potassium bromide pellets using Shimadzu FT–IR– 8400S (400–4000 cm^−1^). The morphological feature of the synthesized nanoparticles were characterized using a ZEISS EVO series SEM model EVO 50 at various magnifications. UV–Vis spectrophotometer (Labman LMSP-UV 1900) was used for the quantitative estimation of the analyte in the range of 300–800 nm. The elemental composition of silver nanoparticles was confirmed using EDX instrument (Zeiss-Sigma Germany).

### 2.3 Sample collection and preparation

**Fig. 1:**
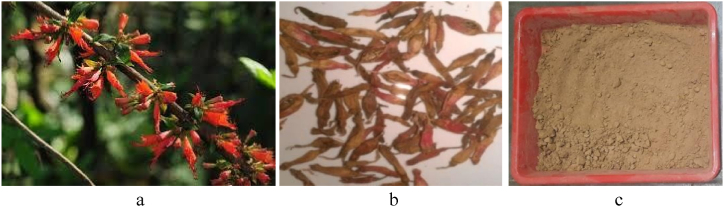
Photographic images of fresh WF (a), dried (b), powdered and sieved WF (c).

The WF was collected from the local field in Coimbatore, Tamil Nadu, India. The photographic images of virgin and processed WF were depicted in Fig. 1 (a-c). The WF was washed and then dried in the shade for 20 days before being rinsed with distilled water. The completely dried WF was powdered and used for the extraction of bioactive compounds.

### 2.4 Isolation of bioactive compounds

10 g of finely powdered WF was placed in a Friedrich condenser. 100 mL of ethanol was taken in a 250 mL round bottom flask and cooled water circulation was enabled in the condenser. The ethanol was refluxed at 80 °C and stirred at 250 rpm for 30 h to ensure the complete extraction of bioactive compounds. During the refluxing process, ethanol vapors reached the distillation arm and condensed into the chamber containing the solid material. This facilitated the dissolution of ethanol-soluble compounds in the solvent. After completing the extraction process, a brownish-yellow solution was obtained and collected in a 250 mL beaker. To maximize the extraction of bioactive compounds, the extraction process was repeated several times. The residue was concentrated by distilling the ethanol, and a small amount of the solvent was kept aside for slow evaporation. Finally, the brownish-yellow crude was collected and stored for further analysis. The same procedure was repeated with methanol at 65 °C and ethyl acetate at 80 °C to obtain the corresponding extracts [29].

### 2.5 Phyto-synthesis of silver nanoparticles

The biogenic synthesis of silver nanoparticles was achieved as per the reported procedure [30]. For this, accurately weight 1.699 g of silver nitrate was dissolved in 1000 mL of distilled water to prepare a 0.01 M silver nitrate solution. From this, 10 mL (0.01 M) solution was mixed thoroughly with different volumes (1–5 mL) of WF extracts using a magnetic stirrer for 30 min and allowed to settle under ambient conditions. The color change to dark brown in the solvent medium indicated the formation of silver nanoparticles. The synthesized silver nanoparticles were centrifuged, and the uncoordinated biological molecules were removed by washing the precipitate thrice using deionized water. Subsequently, the obtained nanoparticles were lyophilized to prevent further oxidation and stored for further analysis.

### 2.6 Preparation of Mueller-Hinton Agar (MHA) medium

A culture medium to facilitate the growth of microorganisms was prepared by dissolving 35 g of MHA in 1 L of distilled water and heated with frequent agitation to ensure complete dissolution. The medium was sterilized in an autoclave at 121 °C for 15 min and then allowed to attain ambient temperature. It was then transferred into sterilized petri dishes uniformly and the pH was adjusted to the required level (7.3 ± 0.1) at 25 °C.

### 2.7 Antibacterial activity

The bacterial inhibition activity of the extracted bioactive compound was evaluated using the agar diffusion method against *E.coli* (gram-negative), *B.cereus* (gram-positive), *K.pneumoniae* (gram-positive) and *S.aureus* (gram-positive) by measuring the zone of inhibition around bacterial growth. For this, bacteria were cultured overnight at 37 °C in MHA. The prepared agar was swabbed with different volumes (10–100 μL) of bacterial cultures (*E.coli, B.cereus, K.pneumoniae, and S.aureus*) using a sterile cotton swab. Subsequently, varying concentrations (10–20 μL) of the virgin extracts were added to separate petri dishes. An antibiotic disc (Moxifloxacin 5 mg) served as a positive control, while ethanol (20 μL) was used as the negative control. The plates were incubated at 37 °C for 24 h. After incubation, the inhibition ability of the samples were analyzed by measuring the zone of inhibition (mm) [31,32]. The experiments were repeated thrice and average values were reported. Similarly, an individual bacterium present in the specimen was inhibited using the synthesized silver nanoparticles (10–25 μL solutions), and their inhibition efficiency was measured at different time intervals (10–60 min) and temperature (25–40 °C). The results were compared with those of the solvent extracts and the positive control.

## 3 Results and discussions

### 3.1 UV–vis spectroscopy

**Fig. 2:**
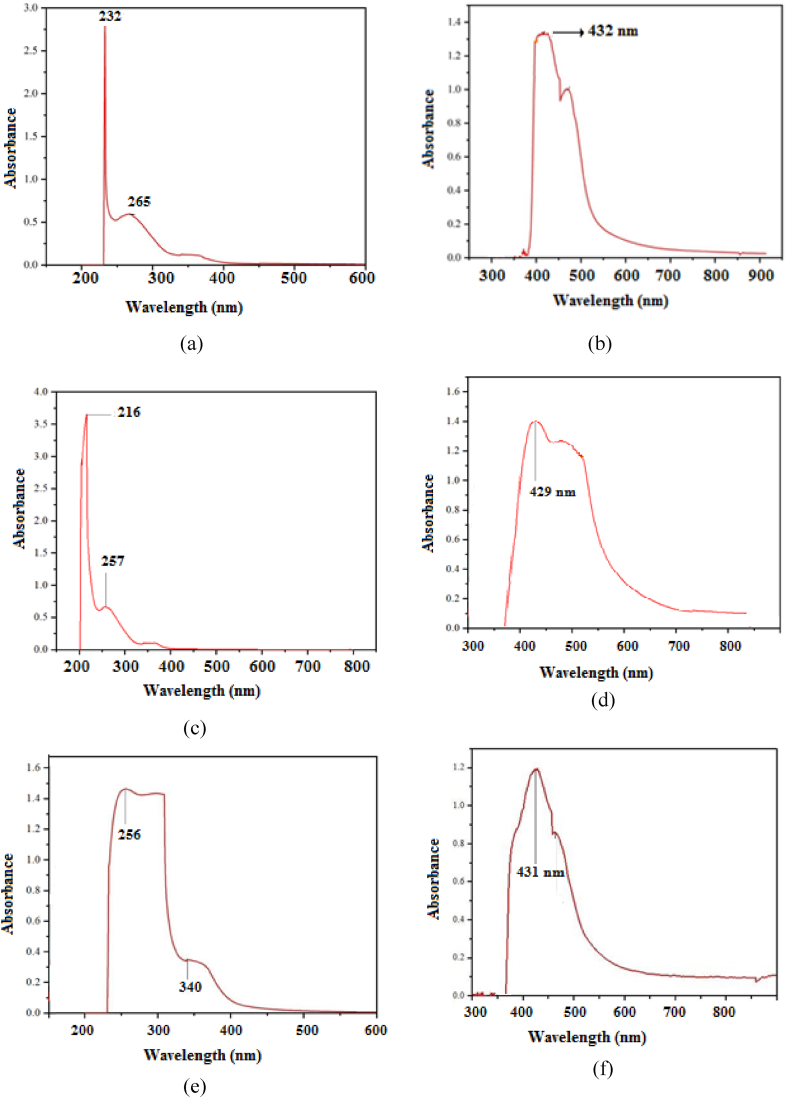
UV spectra of WF extracts of ethanol (a), methanol (c), ethyl acetate (e) and synthesized silver nanoparticles using ethanol extract (b), methanol extract (d) and ethyl acetate extract (f).

A wide range of chemical constituents, including anthraquinone glycosides, flavonoids, tannins, and polyphenols have been isolated from WF [33]. These phytochemicals are known to exhibit significant pharmacological activities. A list of certain reported compounds extracted from WF was presented in Table 1. The representative UV spectra of ethanol, ethyl acetate, and methanol extracts of WF, along with the nanoparticles synthesized using these extracts were displayed in Fig. 2. It has been reported [33] that the solvent extracts of WF contain polyphenolic groups such as lawsone, glucogallin ellagic acid, gallic acid, methyl 3-O-methylgallate, myricetin 3-O-α-L arabinopyranoside, quercetin 3-O-(6-β-galloyl)-β-d- galactopyranoside, quercetin 3-O-α-L-arabinopyranoside, etc. The absorption bands appeared at 232 and 265 nm were attributed to the presence of acid and polyphenolic groups respectively, in the extracted compounds (Fig. 2(a)). The absorption maximum at 216 nm, 257 nm, and 256 nm & 340 nm were due to the presence of conjugated double bonds [34] in the heterocyclic aromatic compounds that present in methanol and ethyl acetate extracts respectively (Fig. 2 (c and e). Similarly, characteristic absorption bands appeared at 432, 429, and 431 nm (Fig. 2(b), (d) and (f)) indicated the formation of silver nanoparticles by ethanol, methanol, and ethyl acetate extracts respectively. An analysis of UV–vis spectra revealed that methanol extract facilitated the reduction of silver ions more effectively than the other two solvents. Furthermore, it was observed that the absorbance intensity increased notably with higher extract concentrations, which can be attributed to the increased concentration of bioactive compounds and the resulting modifications in the shape and particle size of the nanoparticles [35].

**Table 1:**
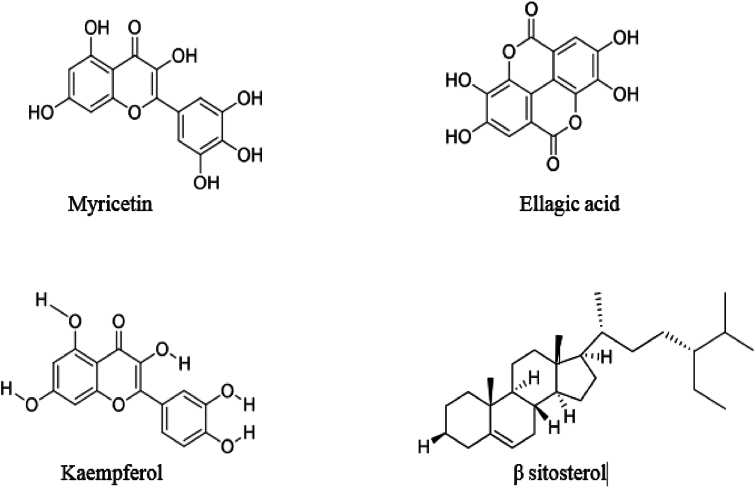
The reported bioactive compounds that present in the WF extract.

### 3.2 FT-IR analysis

**Fig. 3:**
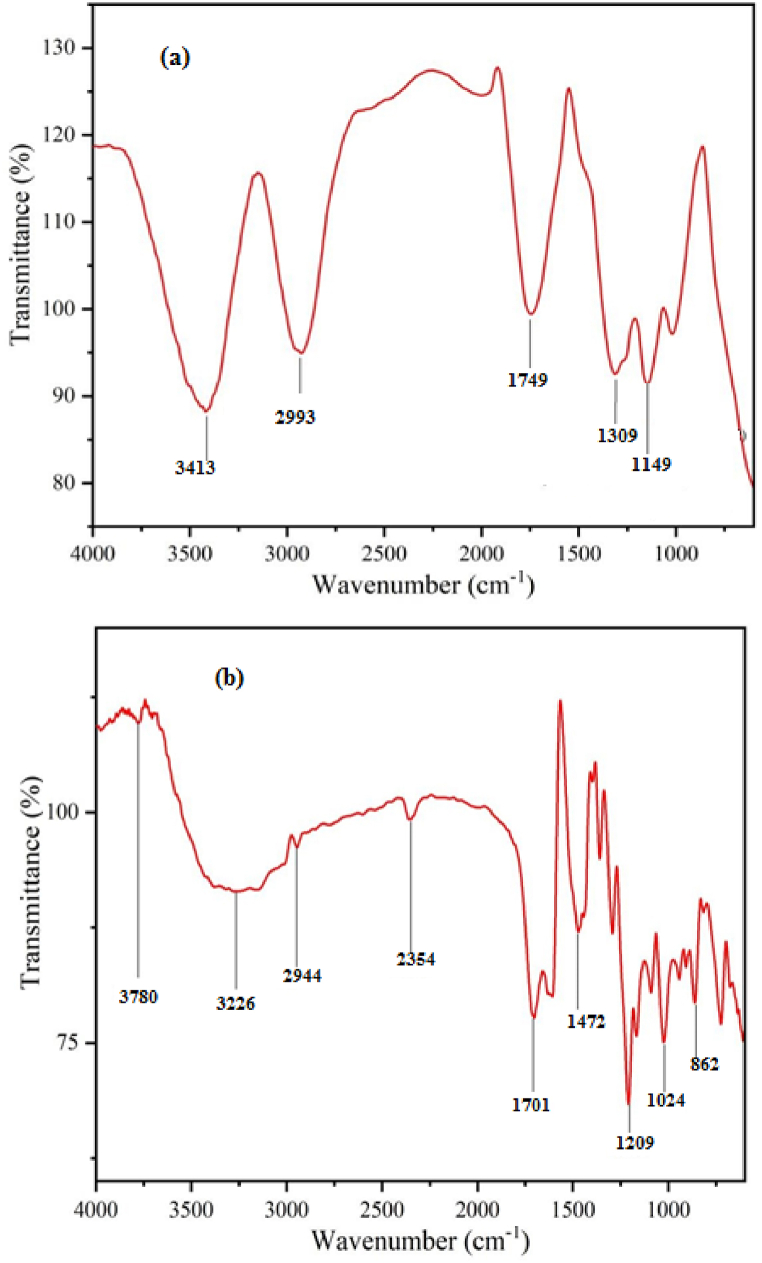
FT-IR spectra of methanol extract of WF without silver nanoparticles (a) and with silver nanoparticles (b).

The FT-IR spectra of the representative dried methanol extract and methanol extract with silver nanoparticles were shown in Fig. 3 (a) and 3(b), respectively. The phytochemical analysis of WF revealed the presence of tannins, anthraquinone glycosides, flavonoids, and polyphenols. The broad absorption bands [36] were observed at 3780 and 3276 cm^−1^, corresponding to O-H stretching vibrations in ring structures and carboxylic acid groups, respectively. The observed band at 2944 cm^−1^ was assigned to C-H symmetric stretching vibration, while the absorption bands [37] at 1701, 1472 and 862 cm^−1^ were due to the C-H bending vibrations, and the C-O stretching vibration was observed at 1209 cm^−1^. Literature reports suggest that [36] β-sitosterol in WF plays a significant role in facilitating the rapid reduction of silver ions to form silver nanoparticles.

### 3.3 Formation of silver nanoparticles and their stability

The introduction of silver ions into solvent extracts of WF facilitated the reduction of these ions into silver nanoparticles. The choice of solvents, such as methanol, ethanol, and ethyl acetate, was due to their efficient ability to extract bioactive compounds from plant parts. Additionally, these solvent extracts facilitated the reduction of silver ions more effectively than other solvents and stabilized the synthesized silver nanoparticles, making them suitable for inhibiting various bacteria [[38], [39], [40]]. This facile synthesis was due to the presence of flavonoids and other bioactive compounds in the solvent extracts. Flavonoids and other bioactive compounds contain multiple hydroxyl groups that were capable of converting silver ions into silver nanoparticles (10–30 nm) by donating the necessary electrons [[38], [39], [40], [41]]. The lower reduction potential of polyphenolic compounds allows for the easy formation of silver nanoparticles through the oxidation of conjugated hydroxyl groups under favourable conditions. In certain cases, the chelation of silver ions by polyphenolic compounds may also facilitate electron transfer. Additionally, these bioactive compounds ensure the stability of the produced silver nanoparticles to the maximum extent [42]. In general, the stability of green-synthesized nanoparticles can be evaluated using UV–Vis spectroscopy and zeta potential measurements [43,44]. In the present study, we conducted UV–Vis spectroscopic measurements over a period of three weeks. The results indicated no significant changes in the spectra recorded each week, suggesting that the synthesized nanoparticles remained stable for the duration of the bacterial inhibition experiments. Further, the size of silver nanoparticles (10–30 nm) was confirmed by the absorption band that appeared around 430 nm in UV–Vis spectra. In the present investigation, the methanol extract produced a higher amount of silver nanoparticles compared to the other two solvents. This can be attributed to the higher yield of bioactive compounds in the methanol extract due to its greater polarity (5.1) compared to the other solvents.

$${Ag}^{+}+1e^{-}\left(one\;electron\;from\;polyphenol\;per\;silver\;ion\right)\;\to Ag+oxidised\;products$$

### 3.4 SEM analysis

**Fig. 4:**
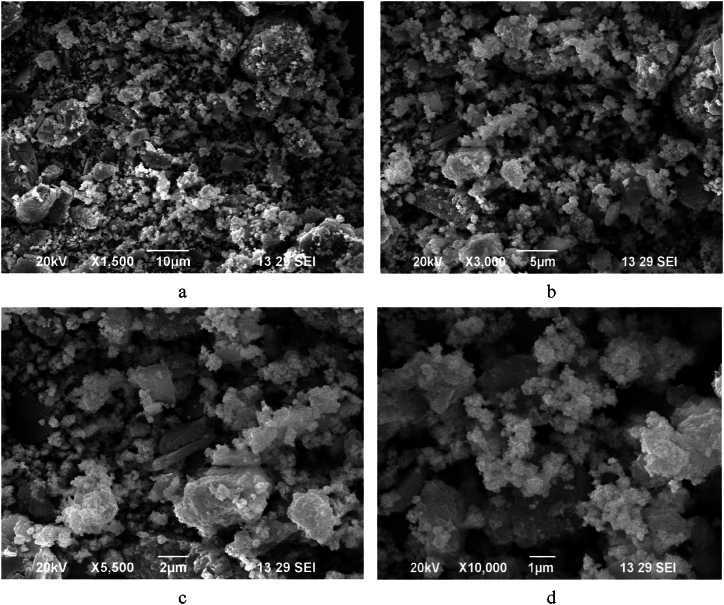
SEM images of synthesized silver nanoparticles using methanol extract of WF.

The representative SEM micrographs of silver nanoparticles reduced by methanol extract of WF at different magnifications were depicted in Fig. 4 (a-d). An analysis of SEM images revealed that the synthesized silver nanoparticles exhibit asymmetric shapes and different particle sizes. along with aggregated morphological features. These findings are consistent with similar results observed for phyto-synthesized silver nanoparticles [45]. Moreover, the nanoparticles produced using the methanol extract demonstrated better-defined shapes compared to those synthesized with the other two solvents. This can be attibuted to the high polarity of methanol over ethanol and ethyl acetate, which enables the extraction of more bioactive compounds. Hence, this facilitated the formation of more silver nanoparticles compared to the other two solvents. Additionaly, the size and shape of silver nanoparticles significantly influence their antibacterial activity. For instance, it has been reported [46,47] that cubic-shaped silver nanoparticles exhibit the highest antibacterial performance, while nanowires demonstrate the weakest inhibition efficiency. Additionally, truncated triangular-shaped particles show a stronger biocidal action compared to spherical and rod-shaped particles. Smaller silver nanoparticles have a greater efficiency because they dissolve more rapidly which can more easily penetrate bacterial cell walls.

### 3.5 EDX study

**Fig. 5:**
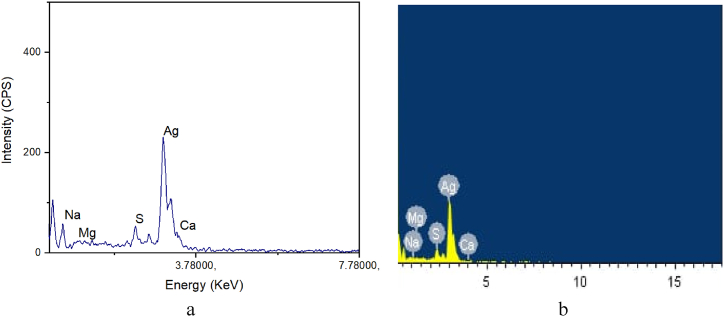
(a) EDX spectrum of silver nanoparticles synthesized using the methanol extract of WF, and (b) EDX image of silver nanoparticles synthesized using the methanol extract of WF.

The EDX spectrum of silver nanoparticles synthesized using methanol extract was presented in Fig. 5 (a & b). The EDX technique was employed to determine the elemental composition of the sample and the relative amounts of each atom in the mixture. In the present study, an analysis of the spectrum revealed that the relative weight percentages of the elements namely Na, Mg, S, Ca and Ag were found to 0.38 %, 0.85 %, 5.35 %, 0.68 %, and 92.74 %, respectively. Similarly, the atomic weight percentages of Na, Mg, S, Ca and Ag were 1.52 %, 5.18 %, 13.26 %, 1.54 %, and 78.50 %, respectively. The results indicated that silver nanoparticles occupied the significant proportion in the spectrum and the synthesized nanoparticles revealed aggregated morphology and non-uniformity in size distribution. Thus, obtained silver nanoparticles exhibited a strong signal at 3 KeV which was due to surface plasmon resonance [[48], [49], [50]].

### 3.6 Bacteria inhibition activity and effects on humans

**Fig. 6:**
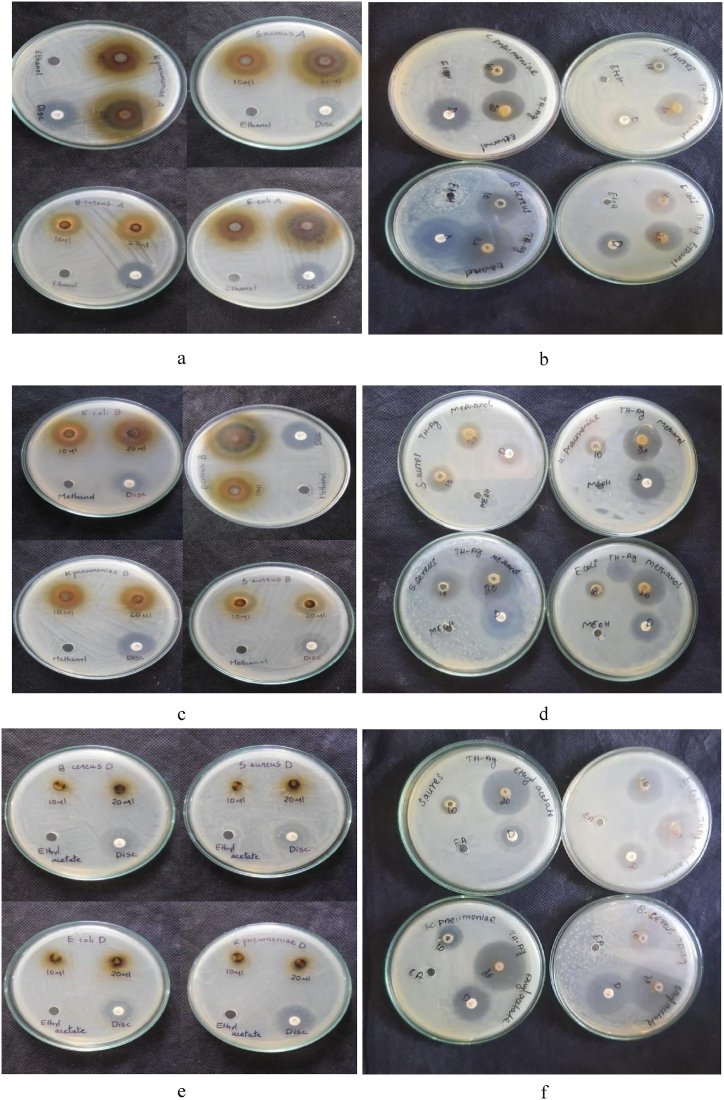
Photographic images showcasing the antibacterial activity of virgin ethanol, its extract and disc (a), virgin methanol its extract and disc (c), virgin ethyl acetate its extract and disc (e), antibacterial activity of phyto-synthesized silver nanoparticles using ethanol extract (b), methanol extract (d) and ethyl acetate (f) against listed bacteria.

The bacterial inhibition efficiency of both solvent extracts and silver nanoparticles synthesized using methanol, ethanol, and ethyl acetate extracts were evaluated against *E.coli* (gram-negative), *B.cereus* (gram-positive), *K.pneumonia*e (gram-positive) and *S.aureus* (gram-positive) bacterial strains. The investigation into the efficacy of solvent extracts and phyto-synthesized silver nanoparticles revealed that the methanol extract and the nanoparticles synthesized using this extract exhibited a larger zone of inhibition compared to the other two solvent extracts and their corresponding solvent-mediated synthesized nanoparticles. This distinctive behavior of methanol was attributed to its higher levels of bioactive compounds and the increased concentration of synthesized silver nanoparticles [51]. The effective zones of inhibition for the solvent extracts and silver nanoparticles against each bacterium were presented in Fig. 6 (a-f). The antibacterial activity of solvent extracts and phyto-synthesized silver nanoparticles were compared with that of commercial antibacterial drug. The results revealed that silver nanoparticles exhibited significantly enhanced inhibition efficiency compared to commercial drug, as detailed in Table 2, Table 3, Table 4. The silver nanoparticles synthesized using methanol and ethanol extracts showed a 0.4–1 fold increase in efficiency against all tested bacteria. However, the silver nanoparticles synthesized using ethyl acetate displayed inhibition efficiency comparable to that of Moxifloxacin.

**Table 2:** Antibacterial activity of methanol extract of WF and silver nanoparticles synthesized using methanol extract.

Virgin methanol extract of WF				
Volume of extract/sample (μL)	Zone of inhibition in mm			
	*E. coli*	*S.aureus*	*B.cereus*	*K.pneumoniae*
10	4	3	4	6
13	5	5	6	5
16	7	7	7	6
20	7	7	7	5
Methanol	0	0	0	0
Disc	5	5	8	5

Silver nanoparticles derived using methanol extract				
Volume of extract and nanoparticles( μL)	Zone of inhibition in mm			
	*E. coli*	*S.aureus*	*B.cereus*	*K.pneumoniae*

10	6	7	5	7
13	7	7	8	6
16	8	8	10	7
20	8	8	10	7
Methanol	0	0	0	0
Disc	5	5	8	5

**Table 3:** Antibacterial activity of ethanol extract of WF and silver nanoparticles synthesized using ethanol extract.

Virgin ethanol extract of WF				
Volume of extract/sample (μL)	Zone of inhibition in mm			
	***E. coli***	*S.aureus*	***B.cereus***	*K.pneumoniae*
10	3	2	3	2
13	4	4	5	4
16	5	5	5	5
20	5	5	5	5
Ethanol		0	0	0
Disc	5	5	8	5

Silver nanoparticles derived using ethanol extract				
Volume of extract and nanoparticles( μL)	Zone of inhibition in mm			
	*E. coli*	*S.aureus*	*B.cereus*	*K.pneumoniae*
10	4	4	4	5
13	5	6	5	5
16	6	8	6	6
20	7	8	7	6
Ethanol	0	0	0	0
Disc	5	5	8	5

**Table 4:** Antibacterial activity of ethyl acetate extract of WF and silver nanoparticles synthesized using ethyl acetate extract.

Virgin ethyl acetate extract of WF				
Volume of extract/sample (μL)	Zone of inhibition in mm			
	*E. coli*	S.aureus	B.cereus	K.pneumoniae
10	1	2	1	1
13	2	2	2	2
16	3	2	3	3
20	3	2	3	3
Ethyl acetate	0	0	0	0
Disc	5	5	8	5

**Silver nanoparticles derived using ethyl acetate extract**				
Volume of extract and nanoparticles( μL)	Zone of inhibition in mm			
	*E. coli*	*S.aureus*	*B.cereus*	*K.pneumoniae*
10	3	5	4	5
13	4	6	5	5
16	5	7	6	6
20	5	7	6	6
Ethyl acetate	0	0	0	0
Disc	5	5	8	5

The antibacterial activity of silver nanoparticles were widely recognized based on their physical characteristics [11,52,53] namely shape, size, mass, binding surface, composition and conditions namely mode of synthesis and pH. The enhanced bactericidal activity of silver nanoparticles cause structural changes in the bacterial cell wall, along with the increased membrane permeability and cell death. Additionally, silver nanoparticles may interact with sulfur and phosphorus rich components such as RNA, DNA, and proteins, which play critical roles in cell division, respiration, and survival. Smaller silver ions can penetrate bacterial cells, causing DNA damage and significantly affecting protein synthesis [54]. The bacterial cell destruction mechanism associated with both solvent extracts and silver nanoparticles may also involve irreversible damage or alteration of membrane permeability, as well as inhibition of bacterial DNA function [55]. The synthesized silver nanoparticles may also induce the production of reactive oxygen species (ROS) in cells [56,57], leading to oxidative stress and cellular damage. Furthermore, ROS can increase cell permeability by damaging membrane lipids, which allows more silver nanoparticles to enter the cell, causing further ROS production. Although silver nanoparticles offer a wide range of antibacterial properties, they can be highly harmful to humans, regardless of their size. For instance, they can affect several human cell lines, including liver cells, human bronchial epithelial cells, and red blood cells [[58], [59], [60]]. In vitro studies have shown that particles with a size of 10 nm are more toxic than larger ones [59]. These nanoparticles can pass through nuclear membrane pores and interact with internal cellular structures [59]. Particles measuring 15 nm can induce acute pulmonary neutrophilic inflammation [60], while 18 nm particles have been reported to cause gender-dependent silver accumulation in the kidney and dose-dependent bile duct hyperplasia in the liver [60]. Additionally, 56 nm nanoparticles can lead to the upregulation of pro-inflammatory cytokines in human lung epithelial cells [60].

### 3.7 Factors influencing inhibition efficiency

**Fig. 7:**
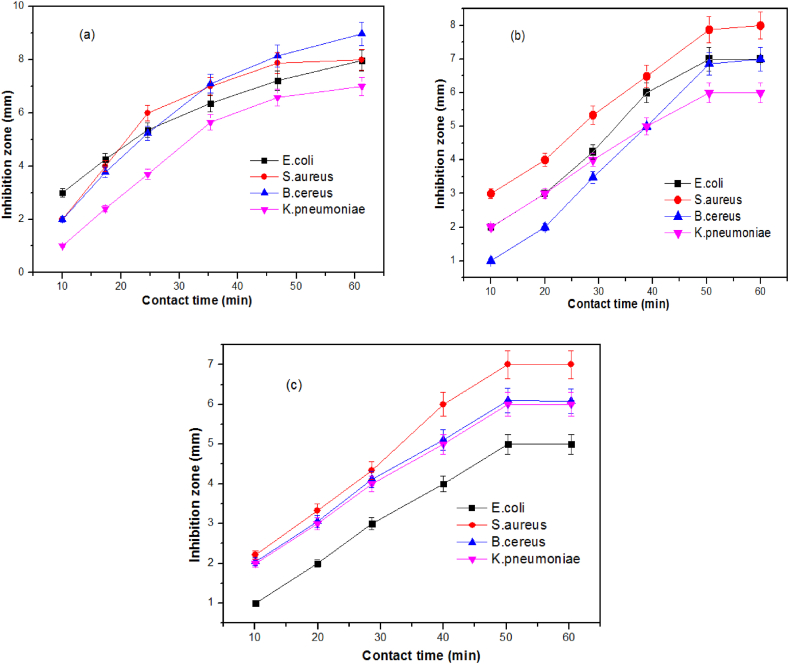
Effects of contact time of (a) methanol extract mediated silver nanoparticles, (b) ethanol extract mediated nanoparticles and (c) ethyl acetate mediated sillver nanoparticles solutions on inhibition efficeicncy

As presented in Table 2, Table 3, Table 4, the concentrations of both virgin solvent extracts and silver nanoparticle solutions significantly altered the zone of inhibition. However, it was noted that a minimum of 10 μL of solvent extracts and nanoparticle solutions were needed to substantially inhibit bacterial growth, which was noted as a minimum inhibitory concentration. However, a maximum of 20 μL of the silver nanoparticle solution and solvent extract were required to achieve the largest zone of inhibition. Beyond this concentration, no significant change in the zone of inhibition was observed for either the solvent extracts or the nanoparticle-containing solutions. In addition, bacterial inhibition efficiency in of the silver nanoparticles synthesized using solvent extracs relation to contact time were presented in Fig. 7 (a-c). The profiles indicate that the maximum zones of inhibition were observed at 37 °C and 50 min of contact time. The reduced zone of inhibition below 30 °C was due to the instability of bacterial growth [61]. Besides, the initial concentration of bacteria was another important factor that could influence the inhibition zone for the chosen volumes of solvent extracts and silver nanoparticles. Analysis indicated that up to 75 μL of agar medium allowed for a maximum zone of inhibition. Beyond this concentration, a reduced inhibition zone was observed due to the increased concentration of bacteria. Upon analysis, it was observed that the minimum bactericidal concentration required to measure the inhibition efficiency of the selected bacteria appeared to be 25 μL.

## 4 Conclusions

The bioactive compounds isolated from WF using ethanol, methanol, and ethyl acetate successfully reduced silver ions into silver nanoparticles. Among these, the methanol extract was particularly effective, producing silver nanoparticles ranging from 10 to 30 nm in size. The antibacterial inhibition efficiency of the extracts, silver nanoparticles, and a commercial drug was evaluated against *S. aureus*, *K. pneumoniae*, *E. coli*, and *B. cereus*. The results indicated that the silver nanoparticles exhibited improved inhibition efficiency compared to the extracts and the commercial drug. The functional groups present in the bioactive compounds were characterized using FT-IR, which confirmed the presence of phenolic –OH and carboxylic acid groups. UV–Vis spectroscopy investigation confirmed the formation of silver nanoparticles, and their elemental composition was analyzed using EDX. Analysis of SEM results further corroborated the formation of silver nanoparticles. Hence, methanol extract may be commercially explored for the synthesis of low-cost silver nanoparticles.

## CRediT authorship contribution statement

**Venkatachalam Dhanapal:** Investigation, Conceptualization. **Pushparaju Subhapriya:** Methodology, Conceptualization. **Karpaganathan Arangarajan:** Writing – original draft, Investigation. **Arumugam Jeevanantham:** Writing – original draft, Investigation. **Perumal Sudhakar:** Validation, Methodology. **Velappan Kandavelu:** Supervision, Formal analysis. **K.S. Nivedhitha:** Software, Investigation. **M.A. Umarfarooq:** Software, Methodology. **N.R. Banapurmath:** Supervision, Conceptualization. **Irfan Anjum Badruddin:** Writing – review & editing, Formal analysis. **Muhammad Nasir Bashir:** Resources, Data curation. **Muhammad Mahmood Ali:** Software, Resources.

## Ethical consideration

The authors have reviewed the Ethics in Publishing guidelines as well as Heliyon's Ethics and Editorial Policies for this research.

## Data availability statement

All the data generated or analyzed during this study are included in this article itself.

## Declaration of Competing Interest

The authors declare that they have no known competing financial interests or personal relationships that could have appeared to influence the work reported in this paper.
